# Outcomes following resective and disconnective strategies in the treatment of epileptic spasms: a systematic review of the literature and individual patient data meta-analysis

**DOI:** 10.3389/fneur.2024.1518554

**Published:** 2024-12-30

**Authors:** Rachel Cottier, Farbod Niazi, Keshav Goël, Catherine Korman, Tiphaine Porte, Thierry Ducruet, Giulia Cossu, Christina Briscoe, Avantika Singh, Chellamani Harini, George M. Ibrahim, Aria Fallah, Alexander G. Weil, Aristides Hadjinicolaou

**Affiliations:** ^1^Brain and Development Research Axis, Azrieli CHU Ste-Justine Research Center, Montreal, QC, Canada; ^2^Division of Neurosurgery, Department of Surgery, Sainte-Justine University Hospital Centre, Montreal, QC, Canada; ^3^Department of Neuroscience, Section of Neurosurgery, University Hospital of Lausanne and University of Lausanne, Lausanne, Switzerland; ^4^Department of Neurosurgery, David Geffen School of Medicine at University of California, Los Angeles, Los Angeles, CA, United States; ^5^Division of Neurology, Department of Pediatrics, Sainte-Justine University Hospital Centre, Montréal, QC, Canada; ^6^Unité de Recherche Clinique Appliquée, Sainte-Justine Hospital, Université de Montréal, Montreal, QC, Canada; ^7^Division of Epilepsy and Clinical Neurophysiology, Department of Neurology, Boston Children’s Hospital, Harvard Medical School, Boston, MA, United States; ^8^Department of Neurology, Division of Pediatric Neurology, Children's Wisconsin, Medical College of Wisconsin, Milwaukee, WI, United States; ^9^Neuroscience and Mental Health, The Hospital for Sick Children, Toronto, ON, Canada; ^10^Division of Neurosurgery, The Hospital for Sick Children, Toronto, ON, Canada; ^11^Institute of Medical Science, University of Toronto, Toronto, ON, Canada; ^12^Division of Neurosurgery, Department of Surgery, University of Montreal Hospital Centre, Montréal, QC, Canada; ^13^Department of Neuroscience, University of Montréal, Montréal, QC, Canada

**Keywords:** epileptic spasms, seizure outcomes, resective surgery, hemisperhrectomy, corpus callosotomy

## Abstract

Epileptic spasms (ES) are a unique seizure type typically presenting in the form of infantile epileptic spasms syndrome (IESS) with characteristic hypsarrhythmia on scalp EEG and a preponderance with developmental delay or regression. While pharmacotherapy is the mainstay of treatment, surgical options, including disconnective or resective procedures, are increasingly recognized as viable therapeutic options for recurrent or persistent ES. However, limited data on safety, effectiveness, and prognostic factors hinder informed decision-making regarding surgery indications, timing, and intervention type. We performed a systematic review and an individual patient data meta-analysis (IPDMA) in accordance with PRISMA guidelines, focusing on surgical interventions for ES and reporting seizure outcomes using the Engel or ILAE scales. Twenty-six studies encompassing 358 ES patients undergoing resection/callosotomy were included. Participants undergoing other approaches (e.g., multiple subpial transections) or multimodality approaches were excluded from analysis. The median age at spasm onset was 6 months (IQR = 3.0–15.6), with a median age at surgery of 37 months (IQR = 17.2–76.8). Most patients (74.1%) exhibited additional seizure types. A total of 136 patients (35.8%) underwent corpus callosotomy (CC), of whom 125 (91.9%) had a complete callosotomy, while 11 (8.1%) had a partial callosotomy. Resective surgery was performed on 222 patients (58.4%). Among those who underwent resection, 109 (49.1%) had both lesional MRI findings and lateralized EEG abnormalities. Overall, 201 patients (56.1%) remained spasm-free at a median postoperative follow-up of 36 months (interquartile range, IQR = 21–60), including 52 (38.2%) from the callosotomy group and 149 (67.1%) from the resective surgery group. In the resective surgery cohort, patients with MRI-confirmed lesions (*p* = 0.026; HR = 0.53, 95% CI = 0.31–0.93) and those who underwent hemispherectomy (*p* = 0.026, HR = 0.46, 95% CI = 0.23–0.91) had better seizure outcomes. Only a minority (24.4%) underwent invasive EEG monitoring prior to ES surgery. Surgical treatment of ES proves effective, with two thirds of patients undergoing resective surgery and a third undergoing CC becoming spasm free. Post-operative developmental improvement was observed in 44 participants (65.7% of those with available data). The presence of lesional MRI and more extensive resection/disconnection (e.g., hemispherectomy) emerged as significant prognostic factors for spasm freedom and can inform clinical decision-making.

## Highlights

- Two thirds of patients undergoing resective surgery for ES become spasm-free
- A third of patients undergoing CC for ES become spasm-free
- Patients with lesional MRI and undergoing larger hemispheric resection are associated with a higher likelihood of achieving spasm freedom following resective surgery.
- Surgical treatment should be considered for patients with refractory or relapsing epileptic spasms.

## 1 Introduction

Epileptic spasms (ES), most often seen in the context of infantile epileptic spasms syndrome (IESS), are a unique seizure type characterized by clusters of ES usually beginning between 1 and 24 months of life, often accompanied by neurodevelopmental stagnation or regression and characteristic electroencephalogram (EEG) findings (1–4). Historically, when those elements were accompanied by the EEG finding of hypsarrhythmia, this clinical triad was referred to as “West syndrome.” However, the term has now evolved into IESS reflecting the need for recognition of this condition without mandatory presence of all elements of the triad (5). Although IESS is rare, affecting approximately 30 to 40 children in 100,000 (6) and with a lifetime prevalence of 1.5–2 per 10,000 children, it remains the most common infantile developmental and epileptic encephalopathy (7, 8). First line pharmacological management, typically with hormonal treatment (e.g., adrenocorticotropic hormone [ACTH], prednisolone) or vigabatrin, results in seizure remission in approximately 50% of the patients (2, 3, 9–12). However, a relapsing disease course is not uncommon, and is a major determinant of encephalopathy (13). Initial primary response to therapy is defined as resolution of ES by day 14 of therapy and lasting at least 28 days (14). Relapse of ES necessitates prompt initiation of standard therapy and is defined as return of a single cluster of spasms, two or more single spasms, or subtle spasm with electrographic correlate following initial primary response (14). While relapse has been reported in 15 to 24% of cases (6), it can occur in up to 66% of children treated with first-line therapy (6, 15). Recently published novel pharmacological regimens have proven to have limited success at completely preventing relapse (16–18), thus highlighting the importance of alternative therapeutic options to mitigate the profound impact of these seizures on neurodevelopment in the setting of ES (6, 19).

While surgical management is a well-established option for other types of pediatric drug-resistant epilepsy (DRE), it has only recently considered as an option for ES that are resistant or relapse following failure of first-line medical treatments (20–22). Recent monocentric clinical studies have shown that resective surgery and CC are effective in reducing the likelihood of ES relapse in certain patient populations. Patients with well-defined lesional ES and concordant data are typically candidates for resective surgery, while those with non-lateralized epileptogenic findings may be considered for CC. However, not all patients with non-lateralized findings, such as those with Aicardi syndrome and agenesis of the corpus callosum, are eligible for CC. Additionally, non-lateralized findings often observed in stroke or hemispheric malformations may make these patients more suitable for resective surgery (20–25). There is a paucity of data on (1) the criteria to select ideal surgical candidates (2), the safety and efficacy of resective/disconnective surgical techniques and (3) prognostic factors in patients undergoing surgical therapy for ES (25). A recent meta-analysis on resective strategies for ES showed good seizure outcomes in most (68.8%), however this study did not report on disconnective procedures which represents at least a third of surgical approaches used in this patient population (26).

Because of the rarity of ES and the majority of published reports originate from single-center studies or RCTs comparing just medical treatments, the ability to draw firm conclusions regarding the optimal therapeutic strategy is limited. Individual Participant Data Meta-Analyses (IPDMAs) utilize patient-level data (27), with the ability to merge existing research and identify patients who stand to gain the most from a specific medical intervention. Prior reports have shown that the insights gained from IPDMAs have been incorporated into medical guidelines, playing a crucial role in the introduction of new approaches into care (28). The primary objective of this IPDMA was to characterize the efficacy/safety profiles and outcomes following disconnective and resective surgical strategies for ES. The secondary objective was to identify the key factors influencing post-operative seizure control (29).

## 2 Material and methods

This research followed the PRISMA (Preferred Reporting Items for Systematic Reviews and Meta-Analyses) guidelines (30, 31). This study was not pre-registered and did not receive any financial support. Institutional ethics approval was not sought since all included data were previously published in the literature.

### 2.1 Search strategy

**Figure 1:**
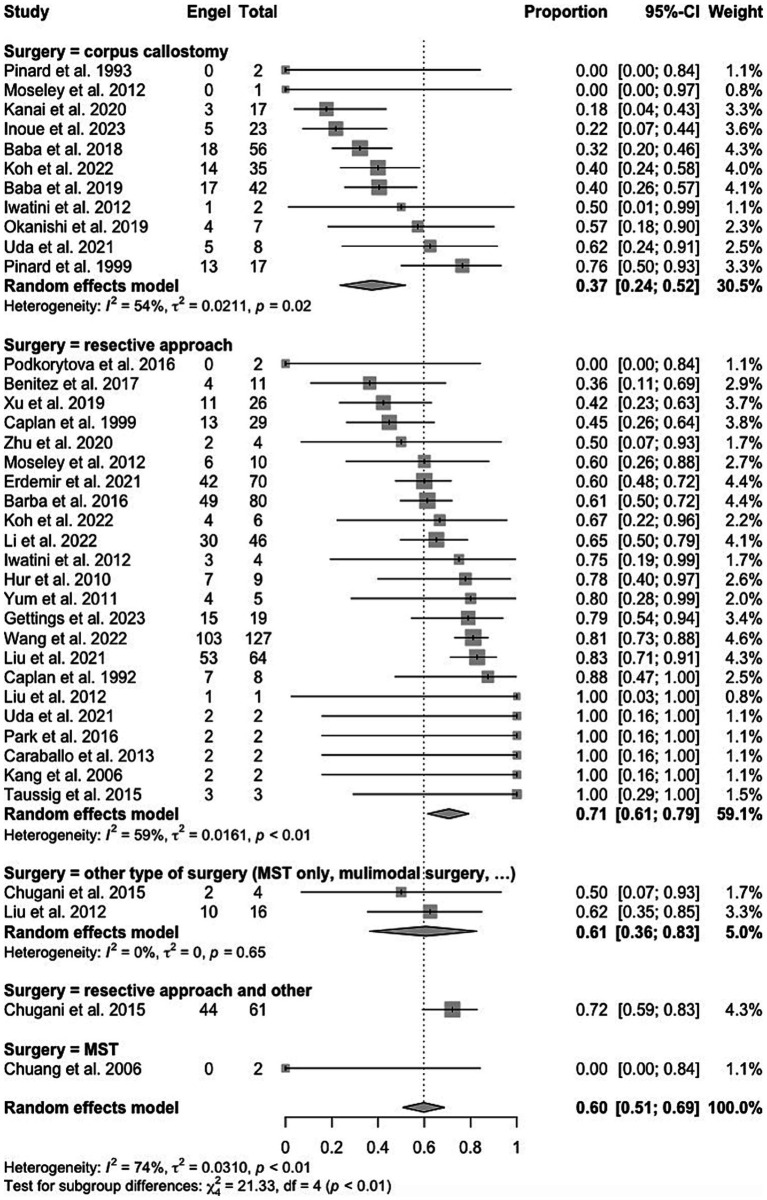
PRISMA flowchart of study selection.

A literature search was performed using the following search terms: “epileptic spasm” and “surgery for epileptic spasm” in Embase, PubMed and Web of Science. PubMed was used as the primary search engine by using free and medical subject heading (MeSH) terms since the other sources did not result in any additional studies. The systematic review included human randomized controlled trials, open-label extension observational studies, retrospective chart studies, and other observational studies published in the English language between January 1990 and September 2023 on the topic of ES surgical outcome. The search strategy is summarized in Figure 1.

### 2.2 Study selection and exclusion criteria

**Figure 2:**
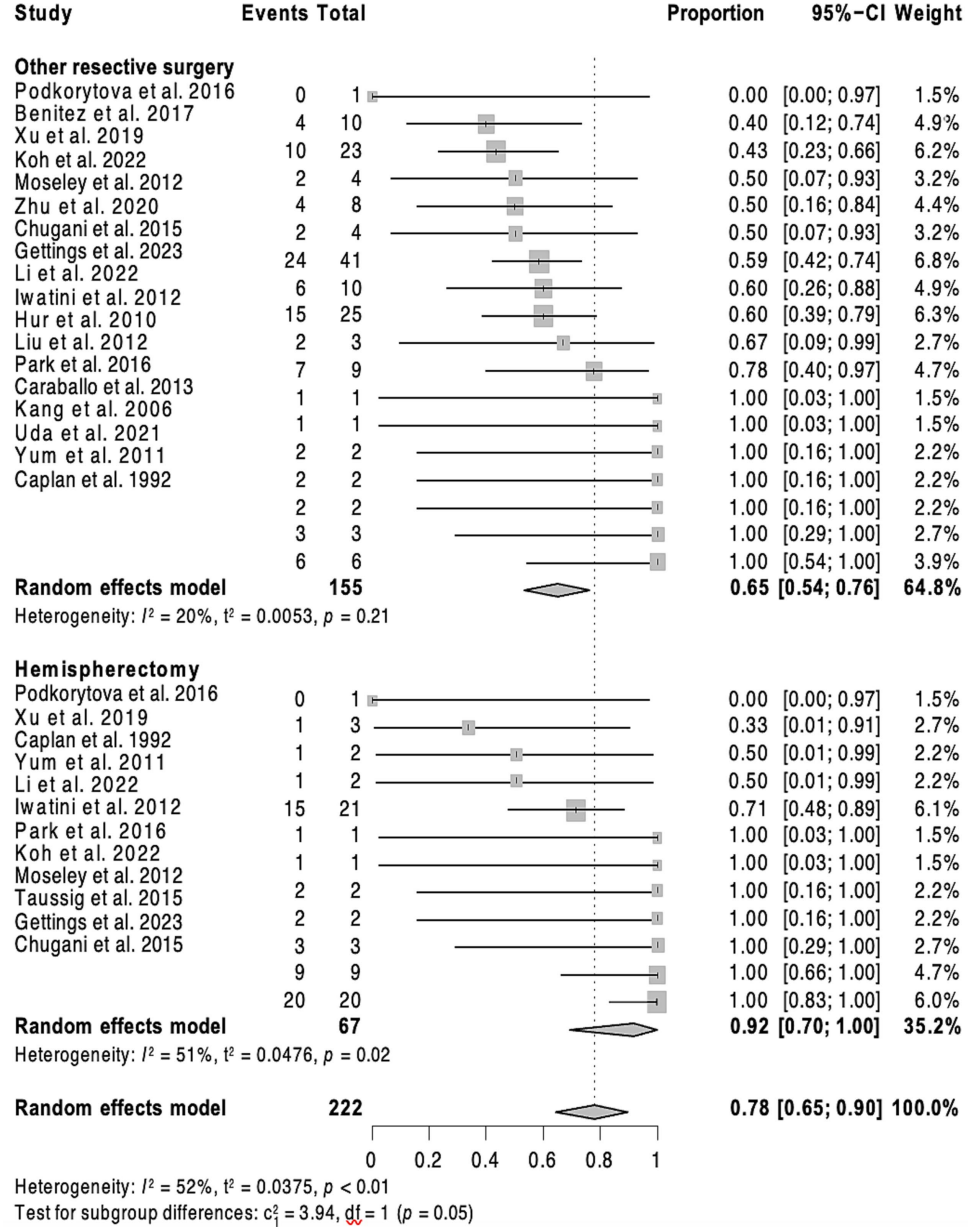
Forest plot showing pooled proportion of overall rate of spasm freedom in patients undergoing corpus callosotomy and resective surgery for epileptic spasms.

**Figure 3:**
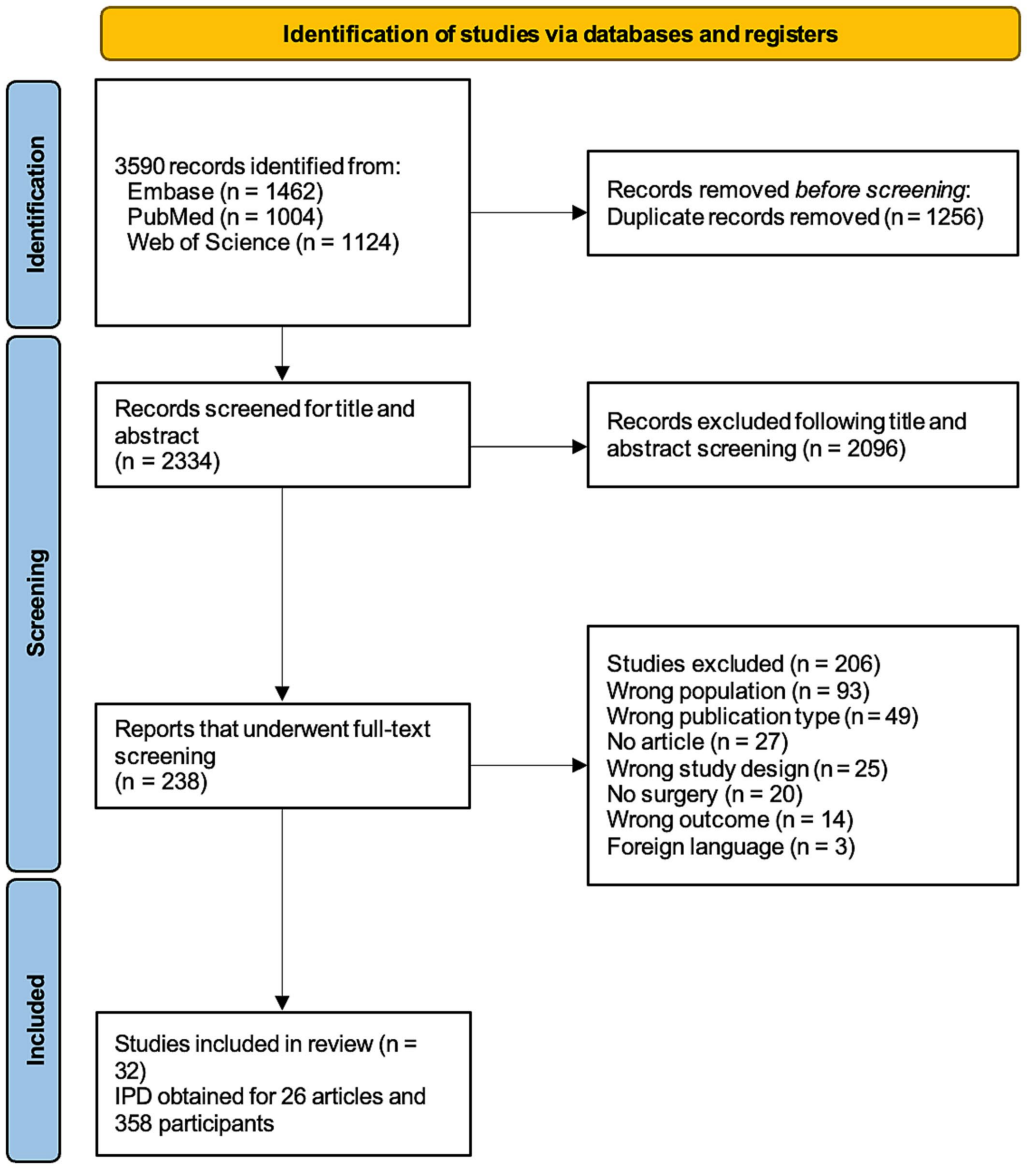
Forest plot showing pooled proportion of overall rate of spasm freedom in patients undergoing hemispherectomy and ‘less than hemispehrectomy” resective surgery.

**Figure 4:**
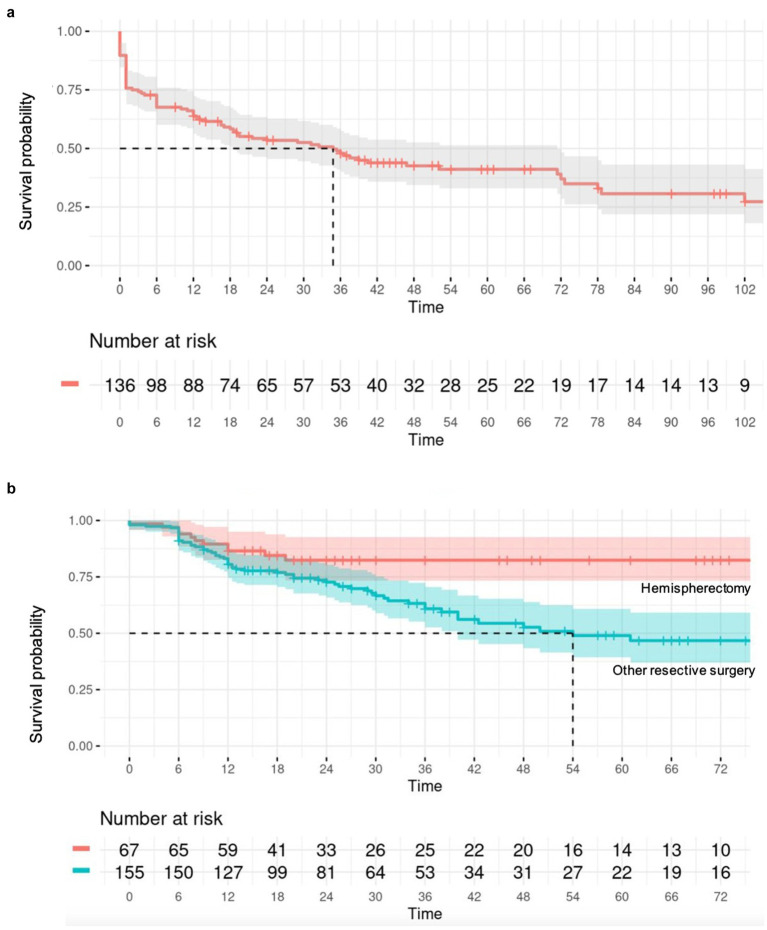
**(A)** Survival analysis illustrated by a Kaplan–Meier curve assessing spasm recurrence in the CC cohort over time (in months); **(B)** Survival analysis illustrated by a Kaplan–Meier curve assessing spasm recurrence in the resective surgery cohort, comparing hemispherectomy to “less than” hemispherectomy resection over time (in months).

All studies presenting their cohort of pediatric patients with ES undergoing epilepsy surgery and describing post-surgical ES outcome with ILAE or Engel classification were included. Patients were required to have a clinical diagnosis of drug-resistant epilepsy (DRE), defined as failure of at least 2 appropriately selected, dosed and tolerated anti-seizure medications. There were no inclusion/exclusion criteria based on clinical or para-clinical (e.g., MRI or EEG) features. Articles of interest were independently examined for title and abstract by two investigators (R.C. and K.G.). Full text of all included studies was then carefully reviewed to assess for eligibility based on pre-determined criteria. Studies with: (1) insufficient information regarding the primary outcome (i.e., spasm freedom), (2) preclinical data (3) a non-English language or (4) individual case reports, were excluded. Furthermore, reference lists were screened to identify possible relevant articles, and a final list of pertinent studies was generated to be included in the review. Twenty-two patients (5.8%) who underwent either multiple subpial transections (MST) or multimodal surgical interventions were excluded from the meta-analysis (Figures 2–4).

### 2.3 Outcome measure

The primary outcome variable was ES outcome, following surgery, assessed using the Engel classification system, which categorizes results into spasm-free (Engel class I) and residual seizure (Engel classes II-IV) (32).

### 2.4 Individual data extraction and quality assessment

Data from all included studies were extracted independently by two investigators (R.C. and K.G.) and stored on a standardized Excel database. When available, IPD from all studies were collected. For studies that did not provide IPD, corresponding authors were contacted to request IPD. Data on patient demographics, preoperative characteristics (e.g., seizure types and frequencies, imaging and EEG findings), type of surgery, length of follow-up, postoperative seizure outcomes and surgery-related complications were collected. Two key variables in this study, namely preoperative imaging and EEG findings were combined to create the following four categories: lesional MRI/lateralized EEG, lesional MRI/non-lateralized EEG, non-lesional MRI/lateralized EEG and non-lesional MRI/non-lateralized EEG. Lesional MRI was characterized by the detection of epileptogenic lesions that could be surgically treated, such as unilateral lobar, multilobar, or hemispheric lesions. Bilateral epileptogenic lesions were also considered surgically treatable if they displayed asymmetry, accompanied by evidence supporting unilateral epileptogenicity, making the patient a candidate for unilateral resective or disconnective surgery. Surgical techniques were subdivided into two main categories, namely resective and disconnective. Resective techniques included focal resection, lobar/multilobar resection and hemispheric surgery, while the disconnective procedures included only partial or complete callosotomies. EEG data were classified as lateralized if any one of the following three criteria was present: (1) hemi-hypsarrhythmia, (2), lateralized slowing, or (3) lateralized epileptiform discharges, either ictal or interictal.

Two investigators (R.C. and K.G.) independently extracted the data of the relevant articles by using a standardized Excel datasheet. Data on study characteristics, patient demographics, preoperative imaging, type of surgery, postoperative seizure outcomes and complications were collected.

For research involving IPD data was obtained directly from original publications. Study authors were contacted in order to obtain any missing IPD. Relevant summary statistics and aggregate data were extracted from published reports and datasets when direct IPD was not available. This included information such as sample size, age at spasm onset, age at surgery, length of follow-up, and the count of Engel I patients.

To ensure data consistency and quality, the data format was standardized across all included studies. This process included addressing missing data, handling outliers, and aligning different measurement scales. The goal was to create a unified dataset to ensure comparability and reliability. The collected data was then securely stored in an Excel file. This evaluation process was important in considering the reliability of the data and ensuring that studies meeting inclusion criteria met the necessary standards.

The following variables were extracted when available: patient demographics, preoperative characteristics (e.g., seizure types and frequencies, imaging and EEG findings), type of surgery, length of follow-up, postoperative seizure outcomes and surgery-related complications. In assessing EEG findings for predicting surgical outcomes, lateralization emerged as a key factor of interest. This encompasses various aspects such as hemi-hypsarrhythmia, lateralized interictal epileptiform discharges (e.g., spikes), and lateralized slowing. EEG findings reported by authors have all been reviewed by epileptologists (33) prior to classification in this study.

In the CC cohort, a comparison was made between patients who had undergone complete and partial disconnections.

### 2.5 Risk of bias assessment

The GRADE framework was applied to assess the quality of every study included in the analysis (34). Additionally, the Newcastle-Ottawa Scale was utilized for assessing potential biases (35). The comprehensive risk of bias for this IPDMA was evaluated by collectively considering the bias risk and quality of all the studies included.

### 2.6 Statistical analysis

The pooled proportion of spasm freedom per unit of time was calculated for all included studies using the inverse variance model, with 95% confidence intervals (CIs) derived through a random-effects model. A Freeman-Tukey double arcsine transformation stabilized variances, and heterogeneity was assessed using I2. Subgroup analysis based on surgical technique was conducted, and publication bias was checked via funnel plots.

The cohort was stratified into two distinct groups: those undergoing CC and those undergoing resective surgery, each pursued with distinct surgical objectives. In the IPD analysis, demographic and clinical data were compared using Chi-squared or Fisher’s exact test, with hazard ratios calculated for continuous variables. Survival analysis employed stepwise Cox regression, with missing data imputed using MICE and Kaplan–Meier curves to visualize time to spasm recurrence. Variables with less than 40% missing data were imputed, while those with more were excluded. A univariate analysis identified potential predictors of outcomes, with a significance threshold of *p* < 0.2. A mixed-effects multivariate analysis was then performed, considering random effects based on study groups, with a significance level of *p* = 0.05 or less. The analysis aimed to identify factors most associated with seizure recurrence, considering covariate interactions and follow-up variability. All analyses were performed using RStudio 2023.06.1 + 524.

## 3 Results

### 3.1 Search results and study selection

All identified patients with surgical outcome for ES were included in this review. A total of 3,590 papers were screened (3,352 studies excluded due to non-related content), 238 full papers were reviewed (206 studies excluded due to missing outcome data). The search strategy resulted in the inclusion of 32 studies, of which 26 presented individual patient data (Table 1, Figure 1). A meta-analysis was carried out on the cohort of 358 patients with individual patient data. Characteristics, quality, and risk of bias of included studies are reported in Supplementary Tables S1, S2.

**Table 1:** Basic data identification of the individual patient data meta-analysis.

Study ID no.	Authors	Year of publication	Country	Type of surgery	Size of sample	Number of Engel 1 patients
1	Inoue et al.	2023	Japan	Disconnective	23	5
2	Gettings et al.	2023	Canada	Resective	19	15
3	Li et al.	2022	China	Resective	46	30
4	Koh et al.	2022	Japan	Resective	6	4
				Disconnective	35	14
5	Uda et al.	2021	Japan	Resective	2	2
				Disconnective	8	5
6	Zhu et al.	2020	China	Resective	4	2
7	Kanai et al.	2020	Japan	Disconnective	17	3
8	Xu et al.	2019	China	Resective	26	11
9	Okanishi et al.	2019	Japan	Disconnective	7	4
10	Baba et al.	2019	Japan	Disconnective	24	7
11	Benitez et al.	2017	United States	Resective	11	4
12	Podkorytova et al.	2016	United States	Resective	2	0
13	Park et al.	2016	United States	Resective	2	2
14	Taussig et al.	2015	France	Resective	3	3
15	Chugani et al.	2015	United States	Resective	61	44
				Other*	4	2
16	Carabello et al.	2013	Argentina	Resective	2	2
17	Iwatini et al.	2012	Japan	Resective	4	3
				Disconnective	2	1
18	Moseley et al.	2012	United States	Resective	10	6
				Disconnective	1	0
19	Liu et al.	2012	China	Resective	1	1
				Other*	16	10
20	Yum et al.	2011	Korea	Resective	5	4
21	Hur et al.	2010	Korea	Resective	9	7
22	Chuang et al.	2006	China	Other*	2	0
23	Kang et al.	2006	Korea	Resective	2	2
24	Pinard et al.	1999	France	Disconnective	17	13
25	Pinard et al.	1993	France	Disconnective	2	0
26	Caplan et al.	1992	United States	Resective	8	7

*Other = MST (multiple subpial transection).

### 3.2 Patient characteristics

The characteristics of the included studies are detailed in Table 1. Study sample size of included studies ranged from 2 to 65 patients. Thirteen studies focused on the resective surgeries, while six included patients undergoing CC exclusively. The remaining seven studies combined both techniques.

Clinical characteristics of the cohort are shown in Table 2. The median age at spasm onset was 6 months (IQR = 3.0–15.6), with a median age at surgery of 37 months (IQR = 17.2–76.8). The majority of patients (*n* = 226, 74.1%) exhibited another type of seizure alongside ES. Among these, focal epilepsy was identified in 80 cases (26.2%), while 18 cases (5.9%) presented with generalized epilepsy. The remaining patients manifested epilepsy of unknown onset or had a combination of both onset types. Epilepsy etiology were available for 311 (86.9%) patients. The most prevalent diagnosis was malformation of cortical development (MCD), identified in 137 patients (44.1%), predominantly involving focal cortical dysplasia (FCD), hemimegalencephaly (HME), and polymicrogyria (PMG). Additional etiologies included 34 cases (10.9%) of tuberous sclerosis complex (TSC), 26 cases (8.4%) of stroke, and 18 cases (5.8%) of tumors. Gliosis alone was observed in 29 patients (9.3%), and 21 patients (6.8%) were diagnosed with West Syndrome without further identified etiology. Nineteen patients (24.4% of the available data) underwent invasive EEG monitoring, consisting of 17 intracranial EEG (Stereo-EEG) procedures and 2 subdural grids. A hundred and 10 patients (84.6% of the available data) received appropriate first-line pharmacological treatment.

**Table 2:** Clinical characteristics of the cohort.

Variable	Value
Total participants, *N*	358
Age at spasm onset, months, *n* = 310	17.1 ± 27.2 (0–156)
Age at surgery, months, *n* = 331	56.1 ± 54.3 (1.8–301.2)
Sex, *n* = 237	
Female	111 (46.8%)
Male	126 (53.2%)
Epilepsy etiology, *n* = 311	
MCD	137 (44.1%)
TSC	34 (10.9%)
Gliosis	29 (9.3%)
Stroke	26 (8.4%)
Tumor	18 (5.8%)
Other	67 (21.5%)
Lesional MRI, *n* = 315	221 (70.2%)
Lateralized EEG, *n* = 267	141 (52.8%)
Hypsarrythmia, *n* = 230	172 (74.8%)
Hemi-hypsarrhythmia, *n* = 230	9 (3.9%)
Lateralized slowing, *n* = 27	12 (44.4%)
Lateralized interictal epileptiform discharges, *n* = 141	111 (78.7%)
Type of surgery, *n* = 358	
Resective surgery	222 (62.0%)
CC	136 (38.0%)
Experienced permanent surgical complication, *n* = 250	30 (12.0%)
Mean length of follow-up, months, *n* = 358	48.5 ± 45.5 (5–322)
Mean time to seizure recurrence, months, *n* = 358	33.6 *±* 33.7 (0–216)
Seizure-free, Engel 1 at last follow-up, *n* = 358	201 (56.1%)

A total of 222 patients (58.4%) underwent resective surgery, encompassing procedures such as hemispherectomy, lobectomy, corticectomy, focal resection, and lesionectomy often referred to as “gross total resection.” Within the resective cohort, 109 patients (49.1%) presented both lesional MRI findings and lateralized (ictal or interictal) EEG results (Table 3). A total of 136 patients (35.8%) underwent CC. The remaining 22 patients (5.8%) underwent either multiple subpial transections (MST) or received multimodal surgical interventions. These patients were excluded from the meta-analysis. The combination of second phase resection to CC concerns 41 patients (11,5%) in this cohort, of which 22 (53.7%) became spasm-free. CC outcomes were significantly better (*p* = 0.03) in patients with non-lesional MRI and non-lateralized EEG where 57.6% achieved Engel 1 outcomes, compared to 34% in other groups (Table 3). The median postoperative follow-up duration was 36 months (interquartile range, IQR = 21–60). The median rate of post-operative complications in the case series was 12.0% (*n* = 30), with hydrocephalus being the most frequently reported complication (*n* = 20, 8.0%).

**Table 3:** Cohort divided by imaging and EEG data.

	Resective surgery (*n* = 222)	Of which Engel 1 (*n* = 149)	Corpus callosotomy (*n* = 136)	Of which Engel 1 (*n* = 52)
Group A	109 (49.1%)	80 (53.7%)	2 (1.5%)	2 (3.8%)
Group B	34 (15.3%)	20 (13.4%)	49 (36.0%)	15 (28.9%)
Group C	27 (12.2%)	18 (12.1%)	1 (0.7%)	1 (1.9%)
Group D	6 (2.7%)	1 (0.7%)	33 (24.3%)	19 (36.5%)
Data unavailable	46 (20.7%)	30 (20.1%)	51 (37.5%)	15 (28.9%)

Group A = lesional MRI and lateralized EEG. Group B = lesional MRI & non lateralized EEG. Group C = non lesional MRI and lateralized EEG. Group D = non lesional MRI and non lateralized EEG.

### 3.3 Heterogeneity

The I^2^ statistic for heterogeneity in our meta-analysis stands at 57% indicating a moderate level of variability across the included studies. This suggests that a substantial proportion of the observed differences in outcomes can be attributed to factors beyond random chance.

### 3.4 Individual patient data meta-analysis

Overall, 201 patients (56.1%) remained spasm-free at a median postoperative follow-up of 36 months (interquartile range, IQR = 21–60), with 52 (38.2%) individuals in the callosotomy cohort and 149 (67.1%) in the resective cohort achieving this outcome. Post-operative developmental improvement was observed in 44 participants (65.7% of those with available data, across a total of 12 studies). Of these, 30 out of 48 participants (62.5%) had undergone resective surgery, and 14 out of 19 (73.7%) had undergone corpus callosotomy. Complication rates were only provided in 16 of the included studies. No discernible predictive factors of seizure outcomes, whether positive or negative, were identified after a multivariate analysis for the callosotomy group. As a result, only the outcomes of the resective surgery cohort are detailed. In the univariate analysis of the resective cohort, factors linked to better seizure outcomes for ES (*p* < 0.20) included undergoing hemispherectomy rather than a more limited resection (*p* = 0.008), having a lesional MRI (*p* = 0.0043), or a history of stroke (*p* = 0.02). Conversely, factors associated with poorer seizure outcomes included having epilepsy of unknown onset (*p* = 0.14), both generalized and focal epilepsy in addition to ES (*p* = 0.017), and being older at the time of surgery (*p* = 0.18). Age at epileptic spasm onset was dichotomized into <2 versus >2 years old but this did not show any significant result. In the multivariate analysis, having a lateralized MRI (*p* = 0.026; HR = 0.53, 95% CI = 0.31–0.93) served as a predictor of seizure freedom after resective surgery. In contrast, having a lateralized EEG, either ictal or interictal, was not associated with a statistically significant impact on seizure outcome. Patients who underwent hemispherectomy (*p* = 0.026, HR = 0.46, 95% CI = 0.23–0.91) experienced a lower likelihood of seizure recurrence compared to those who underwent more limited resection.

## 4 Discussion

Early intervention and seizure control in patients with ES who do not respond to medical therapy is crucial for improving both seizure and cognitive outcomes (23). The classic tenet of epilepsy surgery has more recently been applied to this population, including the surgical removal of the epileptogenic lesion or hemisphere, with or without invasive EEG study, and the performance of CC to palliate spasms or lateralize the epileptogenic focus, to further perform a second stage resective surgery (21). While there is a recent growing scientific evidence supporting the effectiveness and safety surgical strategies for ES, the data is of limited quality and ES are an under-recognized entity. The surgical treatment of this condition has not seen widespread adoption, lagging behind other surgical epileptic conditions. This IPDMA aims to characterize the outcomes and identify predictors of outcomes following surgical therapy for ES to inform decision-making. The main findings of this study are: (1) the majority of patients (68.1%) undergoing resective surgical treatment for ES become seizure-free (68.6% in the study level and 67.1% in the IPDMA), (2) over a third (38.2%) of patients with ES undergoing CC become seizure-free, (3) a lateralized lesion on MRI and a more extensive hemispheric surgery were associated with greater likelihood of seizure freedom in those undergoing resective surgery, and (4) a substantial proportion of patients (*n* = 44, 65.7%) have an improvement in their developmental status after surgery. However, the diversity of assessment tools, including the Bayley Scales of Infant Development and IQ measures, complicates direct comparisons. These instruments evaluate different cognitive domains and may not fully capture the nuanced developmental changes following surgery.

### 4.1 EEG findings

It is important to note that while focal EEG findings are often considered in the surgical evaluation of epilepsy, their relevance to surgical outcomes in patients with ES appears to be limited. In fact, emerging evidence suggests that infants with ES and non-focal EEG patterns may benefit just as much from resective surgery as those with focal abnormalities. Such patients have historically been less likely to be considered for resective surgery. However, our findings align with the growing body of evidence that non-focal EEGs do not predict poorer outcomes in this population, and surgical intervention can still lead to favorable seizure outcomes (36). This highlights the need for a broader consideration of surgical candidacy, regardless of EEG focality.

### 4.2 Corpus callosotomy

This study showed that CC can resolve ES in over a third (38.2%) of patients. CC is a surgical procedure aimed at reducing the spread of seizure activity between hemispheres by severing connections in the corpus callosum. It is particularly effective for generalized seizures such as atonic and tonic seizures, as well as drop attacks (37, 38). In the context of epileptic spasms (ES), CC is used to reduce the frequency and severity of spasms by limiting their bilateral propagation (24). In this study, CC did not resolve ES in two thirds of patients. In these cases, persistent ES are likely due to either focal/unilateral hemispheric ES or an asymmetric bilateral epileptic network (39). Our results suggest CC is more effective for patients with non-lesional MRI and non-lateralized EEG, with over half achieving Engel 1 outcomes. The significant difference (*p* = 0.03) supports the notion that CC may be less effective in patients with lesional MRI or lateralized EEG. The combination of second phase resection after CC can be considered in this setting, which was adopted in 41 patients (11.5%) in this cohort, of whom 22 (53.7%) became spasm-free. This underscores the potential of combining CC and resective surgery to achieve better control of ES, particularly in complex cases where initial surgical measures fail to fully control the seizures. In our study, no significant difference was observed between total and partial callosotomy in terms of seizure outcomes, highlighting the flexibility of both approaches in managing ES.

### 4.3 Resective surgery

This study found that hemispherectomy was associated with a reduced recurrence of seizures compared to other resective surgeries, which was consistent with a previously published report (26). This aligns with existing literature emphasizing the efficacy of hemispherectomy in selected cases of refractory epilepsy with hemispheric involvement (40–42). The presence of a lesional MRI was a favorable predictor of seizure outcome, thus underscoring the importance of integrating MRI findings into the decision-making process for surgical management.

### 4.4 Surgical decision-making in ES

While the International League Against Epilepsy (ILAE) does not currently recommend surgery after the failure of a single antiseizure medication (ASM) specifically for epileptic spasms (ES), the general guidelines for epileptic encephalopathies advocate for early presurgical evaluation following the failure of first-line pharmacotherapy, particularly in patients with identifiable lesions. In our cohort, 110 patients (84.6% of the available data) received appropriate first-line pharmacological treatment (e.g., hormonal therapy or vigabatrin), which typically achieves seizure remission in 50–80% of cases. However, a significant proportion of patients either relapse or fail to respond, emphasizing the need for alternative interventions (2, 3, 9–12). Given the aggressive nature of ES and the significant neurodevelopmental risks associated with prolonged seizures, it is believed that a similar approach should be applied to ES. Our study supports this view, as patients with MRI-confirmed lesions who underwent resective surgery had better seizure outcomes. Therefore, we argue that early and proactive surgical evaluation should be considered for ES patients at onset of drug resistance.

The decision between CC and resective surgery should be informed by specific patient characteristics and seizure localization. While resective surgery showed a higher overall rate of seizure freedom (67.1%), our study also highlights the significant potential of CC, which, alone, completely eliminated seizures in 38.2% of patients despite traditionally being considered a palliative procedure aimed to reducing, rather than resolving, seizures. Thus, CC may be a viable and highly effective option in carefully selected patients with IESS, Lennox–Gastaut syndrome, Lennox-like syndrome (24).

Within the resective surgery cohort, our study found that hemispherectomy was associated with better seizure outcomes compared to other resective procedures (HR = 0.46, 95% CI = 0.23–0.91, *p* = 0.026). Hemispherectomy may be particularly beneficial in cases of widespread hemispheric pathology, where a more extensive resection is necessary to achieve seizure control. On the other hand, for patients with more localized lesions, lesionectomy or lobectomy might be sufficient and preferable due to the lower risk of functional impairment.

### 4.5 Study limitations

Although the use of IPDMA offers significant advantages in terms of statistical power and sample size over single-institution studies, it is not without challenges. A key issue is the moderate heterogeneity observed among the included studies. Some degree of heterogeneity was anticipated due to the diverse nature of the studies included, it is essential to interpret the findings cautiously. Variations in study methodologies, patient populations, and surgical techniques could influence the results. Case reports were excluded to ensure a minimum sample size of two participants per study. However, small sample sizes in some included studies may limit statistical power and reliability. Moreover, while variables with less than 40% missing data were imputed using multiple imputation by chained equations (MICE), the fact that missing data were not randomly distributed may impact the reliability of the results. The analysis also revealed that the proportional hazards assumption in the Cox proportional hazards regression model was not met for certain covariates. This limitation highlights the need for careful consideration of statistical assumptions and potential adjustments in future studies. The study did not include an analysis of neuromodulation techniques, such as vagus nerve stimulation (VNS) or deep brain stimulation (DBS), due to insufficient data availability. Additionally, quality of life (QOL) outcomes following surgery were not evaluated due to the paucity of available reports. These are critical aspects of patient-centered care and should be prioritized in future studies. Moreover, further research is needed to identify the onset of drug resistance, particularly in non-lesional cases. While this study emphasizes the importance of early surgical intervention following the failure of the first ASM, the optimal timing for considering surgical options in non-lesional ES remains uncertain. Further studies should address this gap to refine treatment strategies and improve outcomes for patients with lesional and non-lesional ES. Finally, while the combined cohort of patients had minimal occurrences of complications and deficits, this may result in an underpowered analysis. Future studies should focus on larger cohorts to better assess the frequency and impact of surgical complications.

## 5 Conclusion

This study supports the use of surgical treatment for ES after failure of medical therapy, as most patients undergoing resective surgery and a third undergoing CC are rendered ES free, along with improvement in cognitive outcomes noted after ES surgery. The identification of surgically remediable lesions on MRI and the preference for hemispherectomy over less extensive resective approaches as predictors of seizure freedom can aid in guiding clinical decision-making.

## Data Availability

The raw data supporting the conclusions of this article will be made available by the authors, without undue reservation.
