# Supplementary material for: Outcomes following resective and disconnective strategies in the treatment of epileptic spasms: a systematic review of the literature and individual patient data meta-analysis
Source: Front Neurol. 2024 Dec 30;15:1518554. doi: 10.3389/fneur.2024.1518554 (PMC11726465; doi:10.3389/fneur.2024.1518554)
Supplement: Supplementary file 1 [file Table_1.DOCX]

**Table S1. Characteristics of Included Studies**

| **Authors** | **Year** | **Study Design** | **Country** | **Sample Size** | **Age (at seizure onset)** | **Age (at surgery)** | **Type of Surgery** | **Main Outcomes** |
| --- | --- | --- | --- | --- | --- | --- | --- | --- |
| Barba et al. | 2016 | Retrospective cohort study | Italy | 46 | Mean: 1.3 years | Mean: 5.8 years | Resective approach | Seizure outcome (Engel class) |
| Baba et al. | 2018 | Retrospective cohort study | Japan | 56 | Mean: 5.1 months | Mean: 22.6 months | Corpus callosotomy | Seizure outcome (William’s criteria) |
| Baba et al. | 2019 | Retrospective cohort study | Japan | 42 | Mean: 7.0 months | Mean: 2.8 years | Corpus callosotomy | Seizure outcome (Engel class and William’s criteria) |
| Benitez et al. | 2017 | Retrospective cohort study | United States | 11 | Median: 1 year | Unknown | Resective approach | Level of global disability (Modified Rankin Scale) |
| Caplan et al. | 1992 | Prospective cohort study | United States | 8 | Mean: 3.6 months | Mean: 13.5 months | Resective approach | Non-verbal communication (Early Social Communication Scales) and verbal communication (Bzoch-League Receptive-Emergent Language Scale) |
| Caplan et al. | 1999 | Prospective cohort study | United States | 29 | Mean: 2.7 months | Mean: 18.2 months | Resective approach | Non-verbal communication (Early Social Communication Scales) |
| Caraballo et al. | 2013 | Case study | Argentina | 2 | Patient 1: 12 months  Patient 2: 19 months | Patient 1: 4.5 years  Patient 2: 5.5 years | Resective approach | Epileptic spasms status (EEG recordings), neuropsychological profile (only patient 1), imaging (MRI) |
| Chuang et al. | 2006 | Case study | China | 2 | Patient 1: 14 months  Patient 2: 0 months | Patient 1: 22 months  Patient 2: 3 years | MST | Mean number of daily spasms and more |
| Chugani et al. | 2015 | Retrospective cohort study | United States | 61 (resective)  4 (other) | Mean: 1.08 years | Mean: 5.1 years | Resective approach  Other type of surgery (MST only, multimodal surgery, …) | Seizure outcome (Engel class) |
| Erdermir et al. | 2021 | Retrospective cohort study | United States | 70 | Mean: 6.8 months | Median: 18.5 months | Resective approach | Seizure outcome (Engel class) |
| Gettings et al. | 2023 | Retrospective case series | Canada | 19 | Median: 5 months | Median: 18 months | Resective approach | Seizure outcome (Engel class) and developmental outcome |
| Hur et al. | 2010 | Retrospective cohort study | Korea | 9 | Mean: 7.6 months | Mean: 55.0 months | Resective approach | Seizure outcome (Engel class) and developmental outcomes (Korean Wechsler Intelligence Scale for Children, the Bayley Scales of Infant Development and/or a global assessment scale observed by the patient’s caretakers) |
| Inoue et al. | 2023 | Retrospective cohort study | Japan | 23 | Mean in the post-encephalitis group: 2.8 years  Mean in the non-encephalitis group: 2.9 years | Mean: 8.9 years | Corpus callosotomy | Seizure outcome (Engel class) |
| Iwatini et al. | 2012 | Retrospective cohort study | Japan | 2 (disconnective)  4 (resective) | Mean: 4.7 months | Mean: 1.4 years | Corpus callosotomy  Resective approach | Seizure outcome (Engel class) and developmental prognosis (IQ, language score, ADOS-G) |
| Kanai et al. | 2020 | Retrospective cohort study | Japan | 17 | Mean: 23 months | Mean: 81.4 months | Corpus callosotomy | Seizure outcome (Engel class) |
| Kang et al. | 2006 | Case study | Korea | 2 | Patient 1: 45 weeks conceptional age  Patient 2: 38 weeks conceptional age | Patient 1: 3 months  Patient 2: 4 months | Resective approach | Seizure outcome and developmental status |
| Koh et al. | 2022 | Retrospective cohort study | Japan | 35 (disconnective)  6 (resective) | Mean: 1.7 years | Mean: 5.8 years | Corpus callosotomy  Resective approach | Seizure outcome (Engel class) |
| Li et al. | 2022 | Retrospective cohort study | China | 46 | Mean: 3.00 months | Mean: 15.00 months | Resective approach | Seizure outcome (Engel class) |
| Liu et al. | 2012 | Retrospective cohort study | China | 1 (resective)  16 (other) | Mean: 0.94 years | Mean: 4.23 years | Resective approach  Other type of surgery (MST only, multimodal surgery, …) | Seizure outcome (Engel class) |
| Liu et al. | 2021 | Retrospective cohort study | China | 64 | Mean: 0.95 years | Mean: 3.33 years | Resective approach | Seizure outcome (Engel class) |
| Moseley et al. | 2012 | Retrospective cohort study | United States | 1 (disconnective)  10 (resective) | Mean: 13.4 months | Mean: 39.8 months | Corpus callosotomy  Resective approach | Seizure outcome (Engel class) and developmental quotients |
| Okanishi et al. | 2019 | Retrospective cohort study | Japan | 7 | Median: 3 months | Median: 3 years and 8 months | Corpus callosotomy | Seizure outcome (Engel class) |
| Park et al. | 2016 | Case study | United States | 2 | Patient 1: 4 months  Patient 2: 3 months | Patient 1: 11 months  Patient 2: 3 months | Resective approach | Seizure outcome (Engel class) and neuropsychological evaluations (only patient 1) |
| Pinard et al. | 1993 | Case study | France | 2 | Patient 1: 4 months  Patient 2: 4 months | Patient 1: 4 years  Patient 2: 8 years | Corpus callosotomy | Seizure outcome and developmental outcomes |
| Pinard et al. | 1999 | Retrospective cohort study | France | 17 |  |  | Corpus callosotomy |  |
| Podkorytova et al. | 2016 | Case study | United States | 2 | Patient 1: 10 weeks  Patient 2: 4 weeks | Patient 1: 6.5 years  Patient 2: 1 year and 10 months | Resective approach | Seizure outcome and developmental outcome |
| Taussig et al. | 2015 | Case study | France | 3 | Patient 1: 3 days  Patient 2: 7 months  Patient 3: 3 days | Patient 1: 14 months  Patient 2: 29 months  Patient 3: 7 months | Resective approach | Global disability |
| Uda et al. | 2021 | Retrospective cohort study | Japan | 8 (disconnective)  2 (resective) | Median: 6.5 months | Median: 2.3 years | Corpus callosotomy  Resective approach | Modulation index in each quadrant before and after disconnection |
| Wang et al. | 2022 | Retrospective cohort study | China | 127 | Median: 7 months | 65 patients ≤ 3 years  62 patients > 3 years | Resective approach | Seizure outcome (Engel class) |
| Xu et al. | 2019 | Retrospective cohort study | China | 26 | Mean: 4.2 years | Mean: 8.4 years | Resective approach | Seizure outcome |
| Yum et al. | 2011 | Retrospective cohort study | Korea | 5 | Mean: 5.4 months | Mean: 13.2 months | Resective approach | Seizure outcomes and post-surgery developmental outcomes |
| Zhu et al. | 2020 | Retrospective cohort study | China | 4 | Mean: 15.4 months | Mean: 11 years | Resective approach | Seizure outcome (Engel class) |

**Table S2. Newcastle-Ottawa Scale’s Risk of Bias Tool Evaluation and GRADE Ratings of Articles Included in Meta-analysis**

|  | Newcastle-Ottawa Scale | | | | | | | |  | GRADE |
| --- | --- | --- | --- | --- | --- | --- | --- | --- | --- | --- |
|  | Selection | | | | Comparability | Outcome | | |  | Quality of Evidence |
| Author & Year. | Representativeness of exposed cohort | Selection of non-exposed cohort | Ascertainment of exposure | Demonstration that outcome of interest was not present at start of study | Comparability of cohorts on the basis of the design or analysis | Assessment of outcome | Follow-up long enough for  outcome to occur | Adequacy of follow- up | Total | Certainty Ratings |
| Barba et al. (2016) | * |  | * | * |  | * | * | * | 6 | Low |
| Baba et al. (2018) | * |  | * | * |  | * | * | * | 6 | Low |
| Baba et al. (2019) | * |  | * | * |  | * |  | * | 5 | Low |
| Benitez et al. (2017) | * |  | * | * |  | * | * | * | 6 | Low |
| Caplan et al. (1992) | * |  | * | * |  | * | * | * | 6 | Low |
| Caplan et al. (1999) | * |  | * | * |  | * |  | * | 5 | Low |
| Caraballo et al. (2013) |  |  | * | * |  | * |  |  | 3 | Low |
| Chuang et al. (2006) |  |  | * | * |  | * |  |  | 3 | Low |
| Chugani et al. (2015) | * |  | * | * |  | * |  | * | 5 | Low |
| Erdermir et al. (2021) | * |  | * | * |  | * |  | * | 5 | Low |
| Gettings et al. (2023) | * |  | * | * |  | * | * | * | 6 | Low |
| Hur et al. (2010) | * |  | * | * |  | * | * | * | 6 | Low |
| Inoue et al. (2023) | * |  | * | * |  | * | * | * | 6 | Low |
| Iwatini et al. (2012) | * |  | * | * |  | * | * | * | 6 | Low |
| Kanai et al. (2020) | * |  | * | * |  | * | * | * | 6 | Low |
| Kang et al. (2006) |  |  | * | * |  | * | * |  | 4 | Low |
| Koh et al. (2022) | * |  | * | * |  | * | * | * | 6 | Low |
| Li et al. (2022) | * |  | * | * |  | * | * | * | 6 | Low |
| Liu et al. (2012) | * |  | * | * |  | * | * | * | 6 | Low |
| Liu et al. (2021) | * |  | * | * |  | * | * | * | 6 | Low |
| Moseley et al. (2012) | * |  | * | * |  |  |  | * | 4 | Low |
| Okanishi et al. (2019) | * |  | * | * |  | * |  | * | 5 | Low |
| Park et al. (2016) |  |  | * | * |  | * | * |  | 4 | Low |
| Pinard et al. (1993) |  |  | * | * |  | * | * |  | 4 | Low |
| Pinard et al. (1999) | * |  | * | * |  | * |  | * | 5 | Low |
| Podkorytova et al. (2016) |  |  | * | * |  | * |  |  | 3 | Low |
| Taussig et al. (2015) |  |  | * | * |  | * | * |  | 4 | Low |
| Uda et al. (2021) | * |  | * | * |  | * |  | * | 5 | Low |
| Wang et al. (2022) | * |  | * | * |  | * | * | * | 6 | Low |
| Xu et al. (2019) | * |  | * | * |  | * | * | * | 6 | Low |
| Yum et al. (2011) | * |  | * | * |  | * | * | * | 6 | Low |
| Zhu et al. (2020) | * |  | * | * |  | * | * | * | 6 | Low |
